# European Forum for Primary Care: Position Paper for Primary Care Mental Health: Time for change, now more then ever!

**DOI:** 10.1017/S1463423620000304

**Published:** 2020-12-03

**Authors:** Dineke Smit, Lisa Hill, Ian Walton, Sally Kendall, Jan de Lepeleire

**Affiliations:** 1 PhD Research, Radboud University Nijmegen, Netherlands; 2 Secretary Working Group Mental Health EFPC; 3 Education, Staffordshire University, Stoke, England; 4 Member of Working Group Mental Health EFPC; 5 Educator in Primary Care Mental Health, Manchester, England; 6 Professor, Community Nursing and Public Health, University of Kent, England; 7 Chair Executive Board EFPC; 8 Professor, Medical Department of University Psychiatric Hospital, KU Leuven, Belgium; 9 Chair of Working Group Mental Health EFPC

**Keywords:** EFPC, mental health, primary care

## Abstract

There is a need for a paradigm shift across mental health in primary care to improve the lives of millions of Europeans. To contribute to this paradigm shift, the European Forum for Primary Care (EFPC-MH) working group for Mental Health, produced a Position Paper for Primary Care Mental Health outlining 14 themes that needed prioritizing. These themes were developed and discussed interactively during the EFPC conferences between 2012 and 2019. The Position Paper on Mental Health gives direction to the necessary improvements over the next ten years. The themes vary from preferable healthcare model to the social determinants highlighting issues such as inequalities. The Statement of Mental Health in Primary Care will be established in cooperation with fellow organizations.

## Introduction

The Organisation for Economic Co-operation and Development stated in 2017 that ‘one in ten patients in OECD countries is unnecessarily harmed at the point of care’ (OECD, 2017). Services and processes, the OECD claims, are either harmful or do not deliver enough benefits for patients, including those with mental health problems. In 2018, the OECD, more specifically, stated that better mental health care could improve the lives of millions of Europeans and contribute to stronger economic and employment conditions (OECD, 2018).

The European Forum for Primary Care argues, ‘The majority of health complaints are dealt with through self-care and primary care, delivered in the local community. Addressing the needs of individuals in the context of their families and communities is one of the key features of primary care.’ (EFPC, 2020). Primary care plays an important role in the prevention, detection, and overall improvement of mental health care. The Mental Health working group of the European Forum for Primary Care (EFPC-MH) focuses on ways to improve healthcare policy. The key to this is to share experiences and identify current bottlenecks between national policies and local implementation. A key action of the EFPC-MH is sharing knowledge of what works from service users and clinicians by sharing evidence-based medicine and best practice to stimulate colleagues working in primary care to engage from across Europe to improve mental health care for their communities and themselves. The issues of the former EFPC Statement of Mental Health, from 2006, are partly achieved and needs to be updated.

In 2019, the EFPC-MH wrote a draft Position Statement for Primary Care Mental Health for discussion with the EFPC members and consulted fellow organizations like the European Community-based Mental Health Service Providers Network (EUCOMS), the European General Practice Research Network (EGPRN), and Mental Health Europe (MHE). In 2020, the final EFPC Statement is now published. This article gives an oversight of the development of the statement and its issues.

## Research design

In 2012, the working group EFPC-MH started by listening to what healthcare professionals, social and welfare professionals, and service users indicated about actual day-to-day problems in giving and receiving mental health care in a primary care setting. Subjects such as problematic access to secondary care mental health, lack of research and education in primary care, and social determinants such as poverty and inequality were discussed and were connected to (poor) mental health and its management.

We gained data by semi-structured EFPC workshops annually, allowing us to consult with international attendees, practitioners, and patients, from around 46 countries worldwide. In addition, we gratefully made use of the knowledge obtained from experts during the conferences. We also made use of our sources in between conferences. We also did frequent literature searches to be able to link information to scientific research and/or to available policy information.

We used social research as outlined by Greene (2007) whereupon we had four domains. The first domain addressed philosophical assumptions and different stances. The conferences where we explored this, were attended by students, researchers, professionals, and service users. There were professional views and lived experiences that shaped the work and helped challenge the assumptions. This enabled us to investigate further what was known from those who were best placed and provide a context within Primary Care Mental Health. Within the second domain, we recognized the interconnectedness of what was produced and how this started to give consistent messages. We then took into consideration professional guidelines for practice and by listening to service users we began to understand the need for a paradigm shift on the way that Primary Care Mental Health is understood and delivered. This leads us to the final domain on producing the EFPC position statement, which is our sociopolitical statement, enabling us to have discussions across providers and prioritize which issues and solutions do the most justice to what the parties have shared with us.

Although the whole project was yearly well thought out, at many times, we were surprised by unexpected information, conflicting solutions and judgments, and workshops that had an unexpected dynamic that caused a shift in subject. In these cases, we used an iterative way to interpret what was said and identify the semantics by reflecting on what was captured on worksheets and examining the meaning to gain the perspectives of those attending conferences and workshops in Primary Care Mental Health (Sarantakos, 2005).

## The development of the themes

**2012 Gothenburg** – The authors, four Primary Care Mental Health practitioners and enthusiasts, from Belgium, the Netherlands, and the UK, met whilst each delivering separate presentations at the EFPC conference in Gothenburg. Working with the attendees of our workshops, we were able to issue our first, very brief consensus statement.

‘‘Across Europe, there is a problem with the organisation of mental health care due to siloes, a lack of integration, tribalism, different perspectives between medical, social, and psychological care.”

**2013 Istanbul** – It was found that participants believed that mental health was generally detected well in primary care, however, there was little focus on prevention. There were gaps between primary and secondary care with little integration between health and social care and once in secondary care, the patient’s psychiatric needs were met, but not their social and physical needs.

**2014 Barcelona** – Focused on a common understanding of what we were talking about and look for common understanding and values. Themes produced included a need for educational programs, a consensus on ethical values, and how we measure quality and outcomes.

**2015 Lille** (Primary Care Mental Health Conference) – Recovery was the theme and it was found that it needed to be explored more from a survivor and a professional perspective. This needed to include preparation for life with social skills built-in. Good mental health should be explored and taught at a young age to develop resilience and problem-solving skills.

**2015 Amsterdam** – The need and means for helping, supporting, and managing mental ill-health and trauma of refugee and asylum seekers were explored with strategies for anti-discriminatory practice put forward.

**2016 Riga** – Understanding and explaining complexity in Primary Care was key and this workshop allowed the exploration and a model for management in primary care.

**2017 Porto** – Understanding and discovering major alignment with policy statements from the World Health Organization (Saxena *et al.*, 2006; WHO, 2015) and the United Nations (Pūras, 2017) and defining the major messages concerning the themes produced in the burgeoning position statement.

**2018 Heraklion** – Refining and consulting on the individual areas of the document to develop priorities, consensus, and meaning from what had been produced so far.

**2019 Nanterre** – Presentation of draft finished document for comment, amendment and to share the results of the literature review and by informing the themes produced.

The EFPC Statement of Mental Health initially included 13 themes, first looking at the problems needing to be addressed as brought up by participants in our workshops and then potential solutions. The headings are access, co-creation, complexity, education, inequality, information technology, leadership, a model of care, prevention, research, self-care, spirituality, and workforce development. After presenting these issues in an EFPC webinar in February 2020 and a consultation with EUCOMs on the themes from the position statement, it was decided to add another important heading, Diagnosis. Although it had not come up as a major topic in our workshops, we realized it was important for better joint working with social and secondary care as it helps us to understand why, particularly in Primary Care, the individual is more important than the disease.

## The themes

### Access

Early intervention and support are evidenced to alleviate distress and improve outcomes (Bird *et al.*, 2010; McGorry and Mei, 2018; Read *et al.*, 2018). However, the workshops showed that early intervention is not available in all countries. It was reported that people met barriers and received little or no help until they reach the threshold to meet the criteria to enter services. However, not all receive access to specialized treatment due to their diagnosis or having a comorbidity such as addictions or physical health problems. Access barriers leave people suffering on waiting lists, often unrecognized, misdiagnosed, misunderstood, or ending up in different parts of the system, notably the criminal justice system. Countries with a health insurance funding system reported that treatment was only available for those with symptoms that fitted specific ‘diseases’. Countries without health insurance reported a ‘lottery’ of health care with no consistency of services nor allocation of resources. All countries reported that funding for mental health services is not equal to physical health.

To get a better and fair access to mental health care in a primary care setting, there should be no artificial limits if you require care. Access needs to be at the right time, in the right place, by the right person with the right skills. Primary care can enable swift access at a low level and recognition stage (Dowrick *et al.*, 2016). Collaboration ensures that the patient can get the support they need when they need it, including housing, benefits, work, family, social support, therapy, and specialized mental and physical health services. Primary care offers a continuation of support that matches need, freeing up demand for specialist services. Specialist services need to be working collaboratively with communities that involve patient and caregiver voices, primary care, social care, and the voluntary sector in an integrated system to support the patient on their path to recovery (Woltmann *et al.*, 2012).

### Co-creation

Throughout the workshops, it was voiced that there could be no improvements and decisions made without the patient at the heart of all that was done. Concern was expressed that co-creation is frequently discussed and is represented in international policy documents and is rightly best practice, but, is seldom delivered upon. A systematic review of the literature (Manikam *et al.*, 2017) evidences this view demonstrating the growth of published material from 6 to 150 papers from 2006. However, these are across all of health showing co-creation remains an under-resourced and under-invested area of Primary Care Mental Health. Evidence shows that language and access to some cultural communities are barriers in meeting their mental health needs and co-production could increase participation rates to improve the quality of services (Lloyd *et al.*, 2008; Minogue and Girdlestone, 2010).

To do justice to co-creation patients and caregivers need to be at the heart of all we do, and their voices must be heard within any system change. Integrated pathways of care will work when patient and caregiver experiences are shared, and their world view is understood. The World Health Organization (Murray *et al.*, 1996) emphasizes the need for people-centered health services, which sees people as participants as well as beneficiaries of health care services. Primary Care Mental Health services of the future need to be integrated, responsive, and compassionate in their response. Patients need the education and support they need to make decisions and participate in their care. (World Health Organisation, 2015). This will ensure that services are tailored to meet the breadth and depth of need from local community sources. Investment and mainstreaming of patient-led research and evaluation will benefit services, communities, and individuals alike.

### Complexity in primary care

Mental health is individual with complex biological, social, spiritual, cultural, medical, psychological, existential, and economic factors interconnecting. Clinical guidelines and evidence-based medicine focus on single issues and the best treatment for a sample population that tends to exclude people by age, culture, and gender (Smit and Derksen, 2017). We need to acknowledge the individual, consider comorbidity, and the interconnectedness of all factors. It is time to change systems of care (Sturmberg *et al.*, 2014).

Building teams around primary care to link patients into the community and local mental health services is a good way of ensuring that every individual can be supported to meet their complex needs (Thota *et al.*, 2012). The EFPC underlines that primary care practitioners are experts in complexity, acknowledging the individual, their surroundings, and the interactions between physical and mental health and the need to look after both (Kringos *et al.*, 2010). They are rooted in their communities and in a good position to know about both the problems and the assets within the areas they work in.

### Diagnosis of mental health disorders

The use of psychiatric diagnosis in primary care is problematic (Vanheule *et al.*, 2019). A psychiatric diagnosis misses the individual context of the patient, which is needed to weigh up the symptoms and to answer the request for help (Van Os, 2014). The complaints of the patient are often compounded. Moreover, there is a huge amount of overlap in symptoms resulting in difficulties with classification. A diagnosis does not tell us much about what kind of treatment the patient needs (Allsopp *et al.*, 2019). The professional guidelines, linked to the DSM-5 diagnoses, tend to medicalize mental problems, whereas in primary care contextualized mental health problems are presented and managed.

Instead of focusing on the diagnosis regarding mental health problems, the focus should be much more pragmatic. This pragmatic approach allows recognition of individual experience and gives a better understanding of the distress of the patient (Allsopp *et al.*, 2019). This could be as easy as asking four questions: what happened to you? What is your vulnerability and what is your strength? Where do you want to go? What do you need? (Van Os, 2014; Delespaul *et al.*, 2017). From an integrated care perspective, we advocate for a centralized role for the patient based on their individual needs supported by a whole system approach including local caregivers. Primary Care Mental Health problems should be seen in a context where treatment will stimulate normalization and self-care in harmony with the patient.

### Education

The views were that there is a lack of awareness and skills in communicating and managing well-being and mental health across the whole population, resulting in fear and stigma. The literature supports this view of stigma not just being at a population level but also across professions (Schulze, 2007), education (Martin, 2010), disciplines including physicians (Wallace *et al.*, 2009), communities, and whole countries (Saraceno *et al.*, 2007).

A workforce, fit for the future, requires education that is fit for purpose at all levels. There is a need for education that enables awareness of mental health and well-being from an early age to enable prevention, early detection and to address stigma, the entire population requires a level of knowledge. Research shows that contact combined with education seems to be the most promising avenue (Rüsch *et al.*, 2005; Thompson *et al.*, 2010). Professionally, mental health needs to be embedded across the curriculum of all disciplines starting at an undergraduate level and continuing through post-graduate training and continuing professional development. Co-production and a wider knowledge base that builds on the needs and experience of service users for their recovery journeys (Leamy, 2011; Stuart *et al.*, 2017) is key, as is reflecting people’s cultural and spiritual needs.

### Inequality

Mental health is not discriminatory. We know that certain areas are at higher risk of mental health problems because of greater exposure and vulnerability to unfavorable social, economic, and environmental circumstances (Barnett, 2012; Wilkinson and Pickett, 2017; Ribeiro *et al.*, 2017). Nurture, love, support, and freedom to grow are necessary to develop into a healthy adult, but those born in poorer families and poorer areas have an increased risk of mental health problems in later life (Vannieuwenborg *et al.*, 2015; Tong *et al.*, 2018). There are fewer mental health problems in societies where the population feels they have control over their lives and are involved in decision-making. The poor have less choice and societies are happiest where inequalities are lowest. Poverty divides society and is a major factor for mental illness, the dispossessed lose hope and feel left behind whilst the rich fear losing the security of their wealth.

Inequalities caused by adverse life events, racism, and other forms of discrimination, abuse, violence, neglect, immigration, refugees, asylum seekers, illness, bereavement, relationship breakdown, contact with the criminal justice system and institutional care are all related to an increased risk of mental health problems. There is enough evidence that we should strive for fewer income differences to improve the health and well-being of populations (Pickett and Wilkinson, 2015; Barnett *et al.*, 2012). We will not reduce the stigma associated with mental illness, unless we recognize this and focus on the causes and reduction of their impact, instead of blaming it on inherent weakness in the individual (Clement *et al.*, 2015).

### Information technology

Concern was shared regards the growing shortage of General Practitioners all over Europe. It was voiced that health care systems alone will not be enough to meet the growing need for treatment of mental health problems (Bodenheimer and Smith, 2013). Social media can isolate and be the cause of deterioration of mental health through cyberbullying and social isolation, but it can also be used to benefit mental health by providing education and therapy programs. There is a gap in our knowledge as to the impact of these programs, this gap should be narrowed down.

Information technology has the potential to help those with mental health problems, alongside those who support them. We must make this information technology accessible for everyone, not only for people who can afford it (Allen and Christie, 2016). E-health web-based health interventions have been shown to increase access to care (Hilty *et al.*, 2013). There is evidence of websites, apps and other technologies, which used wisely can be of benefit, particularly to those unable or unwilling to access health and social care services. Guided Internet interventions seem to be cost-effective (Donker *et al.*, 2015).

Information technology cannot replace the human touch; however, it can be used to the benefit of many, either with or without therapist and clinician support. Further research and investment are needed in this area.

### Leadership

To create change, there needs to be governmental, clinical, community, and patient ownership. Within these areas champions for mental health are needed. Like all other conditions presenting in primary care, the approach should be biopsychosocial and existential. ‘There needs to be an integrated approach which is case managed with a recognition that it corresponds with complexity sciences which have cohesion and dynamics in their focus’ (Smit and Derksen, 2015).

The process of change is one that needs guidance and understanding, which requires leadership at all levels. We know ‘one size does not fit all’, so it is important to recognize the uniqueness of each environment and tailor services to meet population needs with leaders to inspire that change. These leaders need to be visible throughout society in schools, faith centers, education, and medicine. Their role is simple to inspire others to create change and develop ownership. Therefore, to enable the rhetoric of national and international statements advocating a change in mental health to become a reality, we need transformational leadership to inspire the change (Hallinger, 2003; Day and Harrison, 2007; Plsek and Greenhalgh, 2001; Plesk and Wilson, 2001; Sturmberg and Martin, 2012; Sturmberg *et al.*, 2012).

### Model of care

Family doctors, from all countries, support people across society and at all stages of their life course. Good primary care provides compassion and cares for the individual, from conception to a good death. There needs to be the recognition that every child, man, or woman has their own unique needs when experiencing mental ill-health. There also needs to be a clear recognition of the families and caregivers, who also have distinct needs that are often not understood in the school, community, and workplace environments.

**Figure 1.**
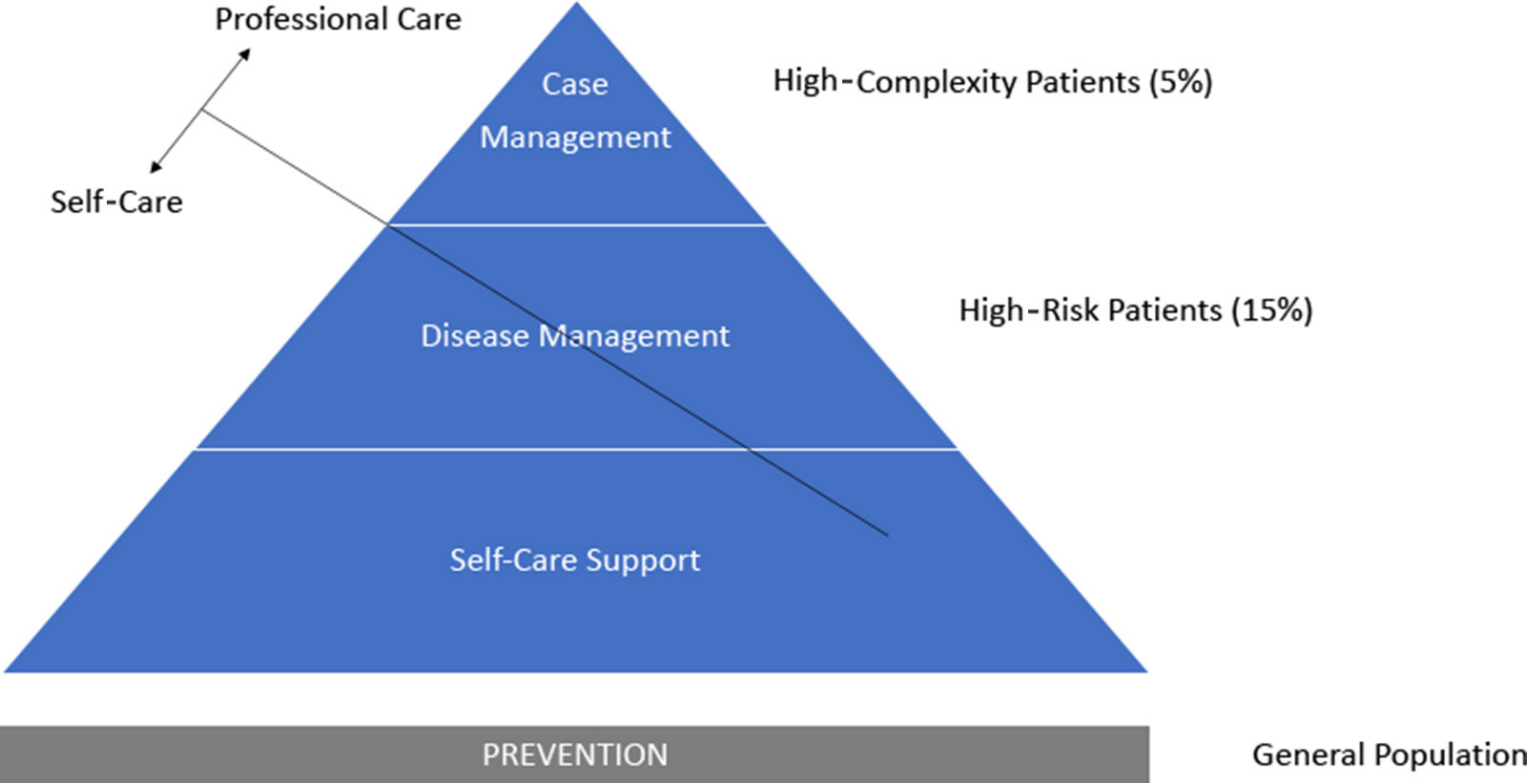
Proposed model of care for mental health based on Kaiser Permanente risk stratification pyramid

Therefore, the EFPC states that integrated working is a priority across community, health, and social care, with best practice examples needing to be understood and shared. Integrated care needs to be at a local level, with skill mix and holistic representation linked to primary care. For more complex needs a dedicated team around the person, offering available support twenty-four hours a day, was identified as key. Mental health needs a clear equitable model of care, which includes investment in prevention, early intervention, access, treatment, and recovery. There is evidence from the USA, Belgium, and the UK that approximately 50% of healthcare costs come from 5% of the population. These high costs, high need patients are often complex and involve multiple agencies (Blumenthal *et al.*, 2016; Rosen, 2018). Adopting a risk stratification model (Figure 1) as a framework to develop a fresh approach for mental health will ensure that investment and integration are key pillars of development. It will also address those more complex patients presenting with comorbidities (Thornicroft *et al.*, 2017). Whilst increasing expenditure on mental health is important it is not the sole driver of change. Improved care and outcomes are equally as important. Reducing inequities in geographic coverage and meeting unmet need means using Primary Care as the first point of support and entry prevents the spiraling of distress and enables the reduction in avoidable hospitalizations. Kaiser Permanente, an American Health Organization, has developed a risk stratification model (Jadad *et al.*, 2010) which has been also used in the UK to categorize levels of care.

‘Case management’ ensures coordination for individuals with complex needs requiring integrated high health and social care support. ‘Care management’, at the next level, provides for high-risk individuals using peer support and education. ‘Supported self-management’ is the level of care for individuals with good control of their mental health in recovery needing only routine medical review. The ‘risk stratification’ allows a framework for both health promotions in the community and for identifying clients at risk.

### Prevention

The European Forum for Primary Care recognizes that the mental health issues presented in primary care are often preventable and can be caused by external and environmental factors (Patel *et al.*, 2010; Hughes *et al.*, 2017). These factors are diverse and include global issues such as war (Lindert *et al.*, 2016; Miller and Rasmussen, 2017), disease (Scott *et al.*, 2009), inequalities (Wilkinson and Pickett, 2017), maltreatment in childhood (Angelakis *et al.*, 2019; Norman *et al.*, 2012), poverty (Patel *et al.*, 2010), and debt (Sweet *et al.*, 2013). Within affluent societies, despite policies and investment, mental health needs are not a priority, are not reducing and in some countries are increasing (Jorm, 2014). Current predictions indicate that by 2030, depression will be the leading cause of disease burden globally (World Health Organisation, 2011) The impact is far-reaching, going beyond a global economic issue, to one which, on a personal, family and community level, impact on people’s spiritual, social, economic, physical, and psychological well-being.

To enable mental and physical health to be of equal status and ensure parity of esteem requires research, evidence, and investment (Sabbe, 2013). The use of education, including mental health promotion, would enable a population approach to manage this ever-increasing problem. Supporting this is the move toward social prescribing in primary care and the use of the community and third sector (Maughan *et al.*, 2016). Therefore, a fundamental paradigm shift toward prevention (Jorm, 2014; Keet *et al.*, 2019) and community-based services including primary care is required (Knapp *et al.*, 2011). This needs to be holistic and compassionate requiring both government strategic support and investment.

### Research

Due to a paucity of accurate data and data analysis regarding Primary Care Mental Health, there is a lack of knowledge and extensive rhetoric at national and international levels that is not well informed. In line with the paradigmatic shift toward an understanding of a praxis of health care that takes account of its complexities, we also need new methods for research (Sturmberg, 2019).

Accurate data will allow the rebalance between self-care and professional care, addressing this data gap. It will inform the redrafting of strategy and policy in Primary Care Mental Health and guide a whole system review for a system that works. It is imperative that research is independent and informed by the patient’s voice, carers and professionals working in communities. Technological solutions also need to be researched as to what works and why, only then can complexity be properly addressed.

#### Self-care

Psychological, social and medical care are available for only short periods, compared to the amount of time that people need to self-care. People are social creatures, for whom altruism, doing things for others is well-evidenced for creating well-being and happiness (Post, 2005; Aknin *et al.*, 2015). Increasingly, particularly in affluent societies loneliness and isolation, known to be detrimental to health (Victor and Yang, 2012; Beutel *et al.*, 2017; Stickley and Koyanagi, 2018; Jessen *et al.*, 2018) and chasing money, rather than happiness, is becoming the norm. Data shows that obesity (Davillas *et al.*, 2016), smoking (Steinberg *et al.*, 2015) and taking drugs (Morley *et al.*, 2015)/and drinking alcohol (Mäkelä *et al.*, 2015) are more common in those with mental health issues, as is lower life expectancy.

Self-care should however not be an excuse for no care. Primary care is in an excellent position to work with patients to create individual lifestyle choices that benefit themselves and the wider community. This will also address issues, such as the negative symptoms normally associated with mental illness, stigma and isolation as well as promoting well-being across the life course. Physical health and mental health are interlinked (Ohrnberger *et al.*, 2017) and health promotion is required at an entire population level. The cornerstone of recovery is hope (Hobbs and Baker, 2012) and underpinning all self-care is that you can recover to be the best you can be.

### Spirituality

We recognize that there are often cultural and spiritual interpretations of mental ill-health, such as black magic, Jinn or juju. The workforce, and society struggle to understand these non-western concepts. They can result not only in isolation and fear for the patient but also for the wider community.

Working with communities to understand their worldview is a vital part of primary care, who are placed in the heart of the community. Recruiting people from different backgrounds with different knowledge bases and adopting a policy of community engagement will enable understanding. Harnessing the knowledge of existing staff and the wider community will also help. Within primary care, there is a growing evidence for the use of Chaplains for Well-being supporting issues such as bereavement and loss (Mowat *et al.*, 2012; Puchalski *et al.*, 2014; Balboni *et al.*, 2014; Kevern and Hill, 2015; Mc Sherry *et al.*, 2016).

#### Workforce development

Within the EFPCMH workshops, patients stated that they feel they are not being listened to. General practitioners and nurses expressed that there is little time to listen and they had not been adequately trained. Primary care staff felt overwhelmed with the volume of mental health and felt that they were left to manage it unsupported, self-reporting burn out and stress and placing pressure on General Practice (Baird *et al.*, 2016).

Mental Health in Primary Care needs to be delivered by a workforce with the skills to assess, manage and treat mental health. This involves developing the interpersonal skills to enable recovery, offering hope and trust. The person needs to be available at the right time, in the right place, offering the right care in the right manner.

Suggestions for new roles in primary care that have been trialed successfully include the role of a social navigator, to navigate the patient through the complexities of the health and social care system (Dohan and Schrag, 2005; Natale-Pereira *et al.*, 2011) and case managers, potentially a generalist role, that proactively supports and coordinates people with mental health problems at a primary care level (Bodenheimer *et al.*, 2002; Wallace *et al.*, 2015) and Chaplains for Well-being (Kevern and Hill, 2015; Mc Sherry *et al.*, 2016).

## Down to work!

To improve mental health care in a primary care setting is an ongoing business. Mental health services in primary care need to develop in a direction whereby they have the capacity and the ability to reach all in our society, particularly those who have the greatest need. The position statement describes the approach that is needed, focussing on the individual, the communities we live in, and services that are integrated. Primary care practitioners with the correct support and training are well-positioned to be central and to coordinate this approach. Research in Primary Care Mental Health remains in its infancy, but if we are to bring mental health services into the 21st century, there is an urgency to invest the means of evaluating the outcomes and ensuring quality as services evolve.

The Norwegian Council for Research (NCR) has funded a research network led by the Centre for Care Research (CCR) in 2018. The Research Unit for General Practice at the University of Bergen, Norway and the European Forum for Primary Care are also partners in this European PRImary care MultiprOfessional Research network (PRIMORE). PRIMORE gave the EFPC-MH working group the opportunity to investigate the relations between mental health care and the connected subjects of education, poverty, and inequality.

Today, the COVID-19 pandemic shows us – again – the urgent need to invest in mental health care. In the policy brief on COVID-19 and mental health issued by the United Nations, Dr Tedros Adhanom Ghebreyesus, Director General of the World Health Organization, noted he is extremely concerned about the impact of the pandemic on people’s mental health (WHO, 2020) not only the patients recovered from the COVID-19 virus, but also family members, frontline health care workers, the elderly due the stay-at-home measures, women, particularly those who are juggling home schooling, working from home and household tasks, and all those who lost their job and/or income. Again, during this corona crisis, most attention went to physical health care, even though mental health should – in all circumstances, be at the core of our humanity, because – as the UN Secretary-General, Antonio Guterres – states ‘it enables us to lead rich and fulfilling lives and to participate in our communities,’ (UN, 2020).

The EFPC underlines again and strongly the statement of Dévora Kestel, Director of the Department of Mental Health and Substance Use at WHO. ‘The scaling-up and reorganization of mental health services that is now needed on a global scale is an opportunity to build a mental health system that is fit for the future.’ (WHO, 2020).
